# On the Use of Machine Learning Models for Prediction of Compressive Strength of Concrete: Influence of Dimensionality Reduction on the Model Performance

**DOI:** 10.3390/ma14040713

**Published:** 2021-02-03

**Authors:** Zhi Wan, Yading Xu, Branko Šavija

**Affiliations:** Faculty of Civil Engineering and Geosciences, Delft University of Technology, 2628CN Delft, The Netherlands; Y.Xu-5@tudelft.nl (Y.X.); B.Savija@tudelft.nl (B.Š.)

**Keywords:** machine learning models, dimensionality reduction, Principal Component Analysis, compressive strength prediction

## Abstract

Compressive strength is the most significant metric to evaluate the mechanical properties of concrete. Machine Learning (ML) methods have shown promising results for predicting compressive strength of concrete. However, at present, no in-depth studies have been devoted to the influence of dimensionality reduction on the performance of different ML models for this application. In this work, four representative ML models, i.e., Linear Regression (LR), Support Vector Regression (SVR), Extreme Gradient Boosting (XGBoost), and Artificial Neural Network (ANN), are trained and used to predict the compressive strength of concrete based on its mixture composition and curing age. For each ML model, three kinds of features are used as input: the eight original features, six Principal Component Analysis (PCA)-selected features, and six manually selected features. The performance as well as the training speed of those four ML models with three different kinds of features is assessed and compared. Based on the obtained results, it is possible to make a relatively accurate prediction of concrete compressive strength using SVR, XGBoost, and ANN with an R-square of over 0.9. When using different features, the highest R-square of the test set occurs in the XGBoost model with manually selected features as inputs (R-square = 0.9339). The prediction accuracy of the SVR model with manually selected features (R-square = 0.9080) or PCA-selected features (R-square = 0.9134) is better than the model with original features (R-square = 0.9003) without dramatic running time change, indicating that dimensionality reduction has a positive influence on SVR model. For XGBoost, the model with PCA-selected features shows poorer performance (R-square = 0.8787) than XGBoost model with original features or manually selected features. A possible reason for this is that the PCA-selected features are not as distinguishable as the manually selected features in this study. In addition, the running time of XGBoost model with PCA-selected features is longer than XGBoost model with original features or manually selected features. In other words, dimensionality reduction by PCA seems to have an adverse effect both on the performance and the running time of XGBoost model. Dimensionality reduction has an adverse effect on the performance of LR model and ANN model because the R-squares on test set of those two models with manually selected features or PCA-selected features are lower than models with original features. Although the running time of ANN is much longer than the other three ML models (less than 1s) in three scenarios, dimensionality reduction has an obviously positive influence on running time without losing much prediction accuracy for ANN model.

## 1. Introduction

Concrete is the most widely used construction material in the world. Among its properties, compressive strength is significant to evaluate its general construction properties for two reasons: first, it is the specification metric most commonly used in engineering practice (e.g., in codes such as Eurocode 2 (EC2) [1] and Chinese code for design of concrete structures (GB50010) [2], and second, other engineering properties (such as Young’s modulus and tensile strength, for example) can be correlated to compressive strength of concrete. Therefore, it is of great importance to make a precise prediction of compressive strength. However, concrete compressive strength is influenced by many factors such as mixture components (water/cement, aggregate, superplasticizers, etc.), curing environment as well as curing age [3,4,5,6]. In practical terms, it would be very beneficial to be able to accurately predict the compressive strength of concrete based on the abovementioned parameters.

At present, measuring the compressive strength of concrete in the lab is crucial for its practical application. However, this is often time-consuming and significant resources are utilized for mixture optimization. In addition, lowering the environmental footprint of concrete production has led to partially/completely replacing cement with supplementary cementitious materials (e.g., fly ash, blast furnace slag, and others [7,8]), further expanding the design space for concrete mix design. Besides, to meet some special requirements of practical engineering nowadays, an increasing number of innovative concrete types (e.g., strain hardening cementitious composites (SHCC), ultra-high performance concrete (UHPC), and self-healing concrete (SHC)) are being developed and need to be investigated in the lab [9,10,11], which makes the prediction of concrete compressive strength based on mixtures increasingly important.

Analytical and numerical models, commonly based on homogenization, are increasingly being used to predict the mechanical properties (compressive/tensile strength, elastic modulus) of concrete [12,13]. In such models, detailed information about the microstructure, spatial distribution, and mechanical properties of individual phases are required [14,15]. In addition, many of these models are computationally expensive, which prohibits their practical applicability. Recently, machine learning (ML) has emerged as a promising way to predict the compressive strength of concrete with high accuracy [16,17,18]. Compared with traditional prediction methods, an advantage of machine learning is that the prediction could be made without knowing the exact relationship between features and compressive strength. Machine learning models have been used for predicting compressive strength of concrete for a long time [19,20]. Different ML models, from simple linear regression to newly developed ensemble methods, have been used to predict concrete compressive strength based on given features. It is reasonable that every ML model has its own advantages and disadvantages. It is of great importance to study the properties of those ML models while predicting concrete compressive strength.

In previous studies, most of the times the learning procedure (hyperparameters tuning) of ML models is not presented and analyzed in detail, making it difficult to assess the validity of the models and replicate the findings. More importantly, most previous studies do not address data preprocessing, a process which can have significant impact on generalization performance of a supervised ML algorithm [21]. For example, dimensionality reduction is crucial for many ML problems, especially for those with many features [22]. However, the influence of dimensionality reduction on the performance of different ML models for compressive strength prediction is currently not investigated.

In this work, we carry out an in-depth comparison between four representative ML models. Among others, Linear Regression (LR) is a parametric regression model used to determine the relation between target (output) and features (input) and it is the simplest regression model [23]. Support Vector Regression (SVR) is a nonparametric ML model which can deal with nonlinear regression problems using kernel function [24]. Extreme Gradient Boosting (XGBoost) is a boosting algorithm based on decision tree [25], and therefore it enables users to analyze the relative importance of features in prediction response. Artificial Neural Network (ANN) is a biologically-inspired method to tackle complex ML tasks with a large number of simple processing elements called neurons [26]. Instead of directly training the ML models with raw data, original features in the dataset are herein first preprocessed with feature selection using mutual information regression (MIR), feature extraction using Principal Component Analysis (PCA) to seek for possible dimensionality reduction. In addition, feature normalization is also carried out to accelerate the training process. The four selected ML models have been trained and tested under three scenarios:

1. Models with original features (i.e., without any feature reduction) to act as a baseline for comparison.
2. Models with reduced dimensionality after PCA, trained and tested with PCA-selected features.
3. Models with the equal number of manually selected features as that in scenario 2.

The performance as well as the training speed of those four ML models under these three scenarios are compared. The influence of dimensionality reduction on the performance of 4 ML models are drawn based on the obtained results.

## 2. Data Source and Theories of Machine Learning Models

### 2.1. Data Source

A set of reliable data is necessary in order to make a precise prediction and comparison between the different ML models. Besides, considering that a small-sized dataset may introduce serious overfitting when using some learning algorithms such as Artificial Neural Network or XGBoost, the size of dataset should not be too small [27]. Therefore, the compressive strength data collected by Yeh [19] with 1030 samples is adopted as our subject. The statistical properties of the dataset are given in Table 1.

### 2.2. Introduction of the Utilized ML Models

Among the ML Models, there are four representative ML models: linear regression (LR), support vector regression (SVR), extreme gradient boosting (XGBoost), and Artificial Neural Network (ANN). In this paper, those ML models are selected to perform the regression and comparison work. A brief introduction about those ML models is given in this part.

#### 2.2.1. Linear Regression (LR)

Linear regression is an old method to evaluate the correlation between two or more features [29]. After deciding the relationship between features (input) and target (output), the learning process will be run to minimize the loss function value (like Mean Squared Error). The parameters that minimize the loss function are exactly the optimal parameters for the regression. Due to its simplicity, the accuracy of this model is not high. The general form of a multiple linear regression model is given in Equation (1) below [30]:

(1) $$\hat{y}\;=\;a_{0}+\overset{m}{\underset{j\;=\;1}{\sum }}a_{j}X_{j}$$

where $\hat{y}$ is the predicted result, $X_{j}$ are the features (input) of the dataset, and $a_{0}$, $a_{1}$, …, $a_{m}$ are the parameters to train.

In this study, this model is employed to fit multiple linear equations that relate the compressive strength and the given features. According to previous research [31], the relation between features and compressive strength is complex and nonlinear. Therefore, in order to improve the prediction accuracy of LR model, polynomial features are created using original features with different polynomial degrees.

#### 2.2.2. Support Vector Regression (SVR)

Support vector machine (SVM) is a powerful and versatile method to deal with the linear/nonlinear classification, regression, and even outlier detection [26]. SVM is a typical kernel method in machine learning. In particular, SVM classifier is aimed to separate different classes with the widest possible street (large margin) between the classes. The linear SVM classifier model predicts the class of a new instance *x* by computing the decision function $W^{T}\cdot x+b\;=\;w_{1}x_{1}+w_{2}x_{2}+\ldots +w_{n}x_{n}+b$. Based on the decision function, prediction is made as Equation (2):

(2) $$\hat{y}\;=\;\{\begin{array}{c} 0,\;W^{T}\cdot x+b<0\\ 1,\;W^{T}\cdot x+b\geq 0 \end{array}$$

The soft margin linear SVM classifier objective is given in Equation (3):

(3) $$\underset{W,b,\zeta }{\min}\frac{1}{2}W^{T}\cdot W+C\overset{m}{\underset{i\;=\;1}{\sum }}\zeta^{\left(i\right)}$$

Subject to $t^{\left(i\right)}\left(W^{T}\cdot x^{\left(i\right)}+b\right)\geq 1-\zeta^{\left(i\right)}$ and $\zeta^{\left(i\right)}\geq 0$ for *i* = 1, 2, …, m, where *W* is the weight vector, $\zeta^{\left(i\right)}$ is a slack variable to measure how much the *i*-th instance is allowed to violate the margin, and *C* is a hyperparameter that allows to define the trade-off between two conflicting objectives: making the slack variables as small as possible to reduce the margin violations, or making $\frac{1}{2}W^{T}\cdot W$ as small as possible to increase the margin.

Support Vector Regression (SVR) is a subset of SVM for regression purposes. Instead of trying to fit the largest possible street between two classes (SVM classifier), SVR tries to fit as many instances as possible on the street while limiting margin violations [32]. Except for linear regression, this algorithm is capable to tackle nonlinear regression by a kernelized SVR. Among the kernels, the Gaussian Radial Basis function (RBF) kernel (Equation (4)) is widely used for its capacity of mapping the original features into infinite dimensionality [28].

(4) $$K\left(a,b\right)\;=\;\exp\left(-\gamma {\left||a-b|\right|}^{2}\right)$$

Hyperparameter *γ* defines how much influence a single training example has and the default value in scikit learn is the reciprocal of the number of features in the dataset [33].

#### 2.2.3. Extreme Gradient Boosting (XGBoost)

Compared with an individual predictor, the model which aggregates the predictions of a group of predictors will often get better predictions. A group of predictors is called an ensemble and this technique is called ensemble learning [25]. There are three different kinds of methods to form an ensemble method, i.e., bagging, boosting, and stacking. For example, Random Forest (RF) is an ensemble of decision trees, generally trained via the bagging method. Unlike bagging, which trains predictors in parallel, boosting trains predictors sequentially.

XGBoost is a scalable end-to-end tree boosting system developed by Chen et al. [34]. It tries to fit the new predictor to the residual errors made by the previous predictor under the gradient boosting framework. The result is predicted using many additive functions as Equation (5) [35]:

(5) $${\bar{y}}_{i}\;=\;y_{i}^{0}+\eta \overset{M}{\underset{k\;=\;1}{\sum }}f_{k}\left(X_{i}\right)$$

where ${\bar{y}}_{i}$ is the predicted result based on features $X_{i}$, $y_{i}^{0}$ is the initial guess (often the mean of the measured values in the training set), and *η* is the learning rate that helps to improve smoothly the model while adding new trees and avoid overfitting.

The estimation $f_{k}$ of the additional k-th estimators is as Equation (6).

(6) $${\bar{y}}_{i}{}^{k}\;=\;{\bar{y}}_{i}{}^{\left(k-1\right)}+\eta f_{k}$$

where ${\bar{y}}_{i}{}^{k}$ is the *k*-th predicted result and $f_{k}$ is defined by the leaves weights.

To learn the functions used in model above, the following regularized objective (Equation (7)) is minimized [35],

(7) $$L\left(\phi \right)\;=\;\underset{i}{\sum }l\left({\hat{y}}_{i},y_{i}\right)+\underset{k}{\sum }\Omega \left(f_{k}\right)$$

where $\Omega \left(f\right)\;=\;\gamma Τ+\frac{1}{2}\lambda {\left||w|\right|}^{2}$.

Here, *l* is a differentiable convex loss function that measures the difference between the prediction ${\hat{y}}_{i}$ and the target $y_{i}$. The second term Ω penalizes the complexity of the model and it acts as an additional regularization term helps to avoid overfitting.

In addition, as XGBoost is based on decision trees and single decision trees are highly interpretable, XGBoost enables users to learn the relative importance or contribution of individual input variables in predicting the response [36]. According to Breimen et al. [37], $I_{l}^{2}\left(T\right)$ could be used as a measure of relevance for each predictor variable $x_{l}$ and is shown in (Equation (8)), in which J is the number of nodes in the tree

(8) $$I_{l}^{2}\left(T\right)\;=\;\overset{J-1}{\underset{t\;=\;1}{\sum }}{\hat{i}}_{t}^{2}I\left(v\left(t\right)\;=\;l\right)$$

The importance measure is generalized to XGBoost by averaged over the trees and is shown in Equation (9), in which M is the number of the trees

(9) $$I_{l}^{2}\;=\;\frac{1}{M}\overset{M}{\underset{m\;=\;1}{\sum }}I_{l}^{2}\left(T_{m}\right)$$

In Scikit-learn, the feature importance is represented with an F score: A higher F score means that the feature contributes relatively more in predicting the response.

#### 2.2.4. Artificial Neural Network (ANN)

Artificial Neural Network (ANN) is a powerful algorithm processing data by simulating the functioning of the biologic neurons [38]. Researchers have carried out a lot of studies on ANN since the 1960s. During the past decades, due to the development of big data and computer power, Deep Learning (DL) has rapidly developed to tackle large and highly complex machine learning tasks [33].

**Figure 1:**
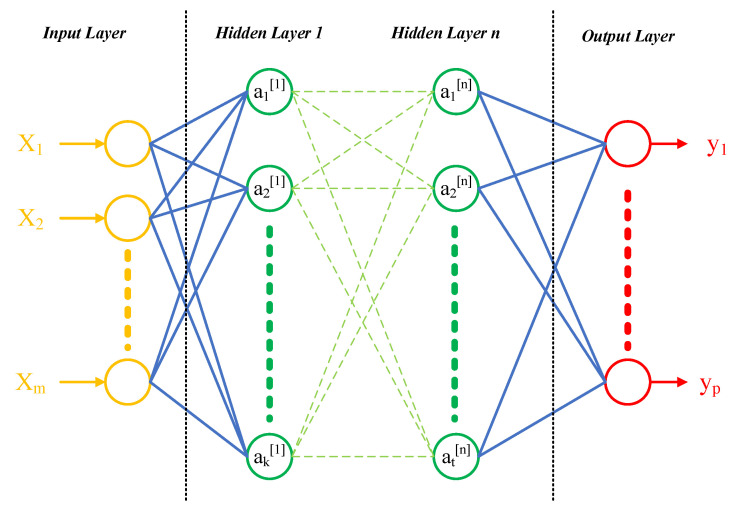
General structure of an Artificial Neural Network (ANN).

ANN uses linking functions to correlate features with targets. There are two processes for ANN: forward propagation and backward propagation. During the forward propagation process, the artificial neuron sums the weighted inputs from the neurons in the previous layer (or input) and then uses activation functions (such as ReLu function, sigmoid function, tanh function, or others) to carry out nonlinear operation [39]. After obtaining the predicted target, the gap between predicted target and actual target is used to improve the weights in each layer, using backward propagation. In the backward propagation process, optimization algorithms such as stochastic gradient descent (SGD), Root Mean square prop (RMSprop), and Adaptive moment estimation (Adam) are employed to improve the weight matrix (W) and bias matrix (b). The two processes continue until the predicted values are close enough to the actual ones (measured by loss function). A general architecture of an ANN is shown in Figure 1 [40]. The calculation of neurons in the *l*-th layer is as Equations (10)–(15).

Forward propagation:

(10) $${\mathbb{Z}}^{\left[l\right]}\;=\;W^{\left[l\right]}A^{\left[l-1\right]}+b^{\left[l\right]}$$

(11) $$A^{\left[l\right]}\;=\;g^{\left[l\right]}\left({\mathbb{Z}}^{\left[l\right]}\right)$$

Backward propagation:

(12) $$d{\mathbb{Z}}^{\left[l\right]}\;=\;dA^{\left[l\right]}*{g^{\left[l\right]}}^{\prime }\left({\mathbb{Z}}^{\left[l\right]}\right)$$

(13) $$dW^{\left[l\right]}\;=\;\frac{1}{m}d{\mathbb{Z}}^{\left[l\right]}*{A^{\left[l-1\right]}}^{T}$$

(14) $$db^{\left[l\right]}\;=\;\frac{1}{m}\sum_{i\;=\;1}^{m}d{\mathbb{Z}}^{\left[l\right]}\;(\mathrm{axis}\;=\;1)$$

(15) $$dA^{\left[l-1\right]}\;=\;{W^{\left[l\right]}}^{T}*\;d{\mathbb{Z}}^{\left[l\right]}$$

where $W^{\left[l\right]}$, $b^{\left[l\right]}$ are the weight matrix and bias matrix of layer *l*; ${\mathbb{Z}}^{\left[l\right]}$ is the weighted sum matrix of layer *l*; $A^{\left[l\right]}$ is the activation of units in layer *l*; $A^{\left[l\right]}\;=\;\left[a_{1}^{\left[l\right]},a_{2}^{\left[l\right]}\cdots ,\;a_{k}^{\left[l\right]}\right]$, $g^{\left[l\right]}\left(x\right)$ is the activation function of layer *l*; $d{\mathbb{Z}}^{\left[l\right]}$, $dW^{\left[l\right]}$, $db^{\left[l\right]}$ are the derivative of the weighted sum matrix ${\mathbb{Z}}^{\left[l\right]}$, weight matrix $W^{\left[l\right]}$, bias matrix $b^{\left[l\right]}$ in layer *l*, respectively; and $dA^{\left[l-1\right]}$ is the derivative of activation of units in layer *l* − 1.

## 3. Prediction Process with Different ML Models

### 3.1. Data Preprocessing

Before feeding the dataset into the ML models, it is necessary to preprocess the data to accelerate the learning process [21]. As the dataset we use is relatively complete and clean, we only perform feature selection, feature extraction and feature normalization sequentially.

#### 3.1.1. Feature Selection and Extraction

In Machine Learning applications, identifying the most characterizing features is crucial to minimize the cost function. In this work, mutual information regression (MIR) [41] is first employed to detect the dependency of the original features. In MIR, the mutual information of two given variables is defined in terms of their probabilistic density functions *p*(*x*), *p*(*y*), and *p*(*x*,*y*) (Equation (16))

(16) $$I\left(x,y\right)\;=\;\iint^{\;}p\left(x,y\right)log\frac{p\left(x,y\right)}{p\left(x\right)P\left(y\right)}dxdy$$

The selected features $x_{i}$ are required to have the largest mutual information. In terms of sequential search, the m best individual features are often selected as the m features.

After analysis with mutual information in scikit-learn, the number of selected features is still 8, which is the same with the number of original features. In other words, none of the original features could be directly abandoned without losing much information. It is likely that the prediction models will have good prediction results if all the original features are retained in this study.

Considering that it is not suitable to reduce dimensionality by directly abandoning original features, Principal Component Analysis (PCA) is employed to extract the original features. In PCA, the features are extracted by projecting the original vector to a lower dimensional latent space and find a new set of coordinates to define the original vectors. In Scikit-learn, singular value decomposition (SVD) (Equations (17) and (18)) is utilized using the Numpy package [42] to perform the Principal Component Analysis [43],

(17) $$\tilde{x}\;=\;x-x\_mean$$

(18) $$\tilde{x}\;=\;U\Sigma V^{T}$$

where x_mean is the mean values of *x*, $\Sigma^{T}\Sigma$ matrix contains the eigenvalues that determine the variance explained in each principal component, while the $V^{T}$ matrix contains the principal components in order of decreasing variance explained.

In this work, PCA is performed to detect the possible dimensionality reduction by analyzing the cumulative explained variance. When the cumulative explained variance approaches 1, it means that an increasing amount of information in the dataset is retained. All information in the dataset is retained when the cumulative explained variance equals to 1.

**Figure 2:**
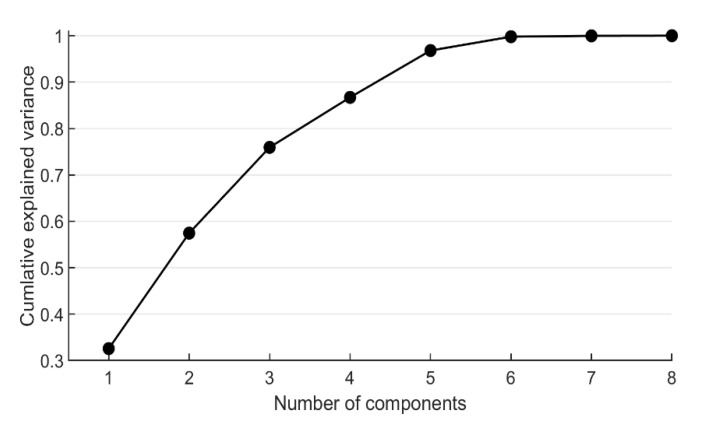
Cumulative explained variance ratio with respect to number of dimensions (PCA).

Based on the obtained results from PCA, the explained variance ratio of each selected component is shown in Table 2. The relation between cumulative explained variance and the number of components after dimensionality reduction is shown in Figure 2.

From Figure 2, it is noted that the cumulative explained variance is lower than 1.0 when the number of components is less than 8, indicating that the 8 features are more or less related with each other. The results from PCA are in good agreement with that of the result of MIR. However, when the number of components is reduced to 6 by PCA, the original information is well reserved (cumulative explained variance is 0.997895). Considering that the total number of components is not too large, models using the 8 original features, 6 PCA-selected features, and 6 manually selected features are used as the input for the ML models in this study.

#### 3.1.2. Feature Normalization

Another common preprocessing method in ML is feature normalization, which could also accelerate the learning process. From Table 1, it can be seen that the ranges of features are quite different: for example, mixtures contain on average 6 kg/m^3^ of superplasticizer, but more than 970 kg/m^3^ of coarse aggregate. It is known that ML models, which are smooth functions of the inputs, are affected by the scale of the input [44]. Therefore, feature normalization, where the mean values and the variances of each feature are 0 and 1, respectively (Equation (19)), is adopted to deal with the dataset.

(19) $$x^{\prime }\;=\;\frac{x-\bar{x}}{\sigma }$$

where $\bar{x}$ and σ are the mean and the standard deviation of feature x, respectively.

### 3.2. Prediction Process with Original Features

To avoid data leakage, the original dataset is first split with a split of 70–30% between the training and test sets, i.e., trainset (size = 721) and test set (size = 309). Among others, the training set is used to train the ML models while the test set is used to verify the performance of ML models (generalization ability). As the compressive strength prediction is a regression task, coefficient of determination (R-square) is selected as a metric to evaluate the accuracy of ML models. In addition, considering that the gap of root mean squares error (RMSE) or mean absolute error (MAE) between different ML models is not as distinguishable as mean squared error (MSE), MSE is also employed to analyze the performance of 4 ML models with 3 different kinds of features. The mathematical formulations of R-square and MSE are given in Equations (20) and (21) [40]:

(20) $$R^{2}\left(y\;,y^{\prime }\right)\;=\;1-\frac{\sum^{\;}{\left(y_{i}-y_{i}{}^{\prime }\right)}^{2}}{\sum^{\;}{\left(y_{i}-\bar{y}\right)}^{2}}$$

(21) $$MSE\;=\;\frac{1}{n}\sum_{i\;=\;1}^{i\;=\;n}{\left|y-y^{\prime }\right|}^{2}$$

where $y_{i}$ and $y_{i}{}^{\prime }$ are the actual and predicted values of the *i*-th sample, and $\bar{y}$ is the mean value of compressive strength.

Linear Regression, Support Vector Regression, and Extreme Gradient Boosting are implemented under the framework of scikit-learn [33]. The ANN model is implemented under the framework of Keras [45]. Since there is no need to know the relation between features and targets in advance (except in the LR model), the learning process mainly focuses on tuning hyperparameters and obtaining the optimal parameters for each ML model.

#### 3.2.1. Linear Regression (LR)

In linear regression, the selection of the regression hypothesis is crucial. Except for the linear combination of 8 original features, different polynomial features are also created to look for the optimal performance of LR model on the test set. However, according to Equation (22), the number of polynomial features dramatically increases with the polynomial degree. When polynomial degree is 5, the number of polynomial features is 1286, which is even larger than the data size (1030), and the model will be severely overfitting. Therefore, polynomial regressions with degree varying from 1 to 4 are performed:

(22) $$N\;=\;\frac{\left(n+d\right)!}{d!n!}-1$$

where *N* and *n* are the number of new features and original features, respectively, and *d* is the polynomial degree.

The prediction process is simple because no hyperparameters are tuned in this work. The result of polynomial regression with different degrees is shown in Table 3.

**Figure 3:**
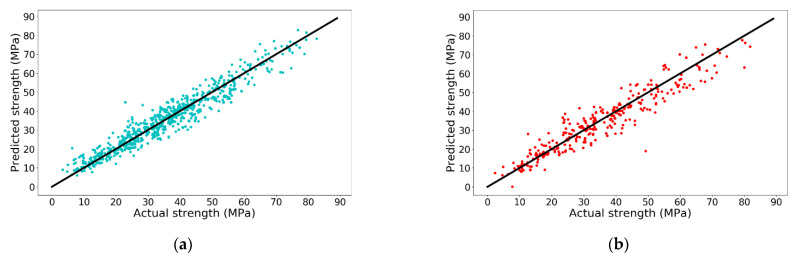
Prediction accuracy of 3rd degree polynomial LR model on (**a**) training set and (**b**) test set.

From Table 3, it is noted that when polynomial degree increases from 1 to 3, R-squares of both training set and test set increase. However, when the polynomial degree continues increasing to 4, the R-square of training set increases while the R-square of the test set sharply decreases. A negative R-square indicates that the predicted result from ML model is even poorer than directly using the mean value of the test set as the predicted values for all samples in test set, indicating the model is severely overfitting. In other words, the performance of LR model with 496 features on the test set is the worst among those LR models. Therefore, R-square of model with 164 features (polynomial degree = 3) on the test set is the highest with a value of 0.8914 and the accuracy is shown in Figure 3.

From Figure 3, the performance of LR model with polynomial degree of 3 is remarkable in both training set and test set. However, LR model only works with positive integer polynomial features, which significantly restricts its further improvement on prediction accuracy. For example, LR model is not able to map water/cement ratio as a polynomial feature because cement is a feature with a polynomial degree of −1 in water/cement ratio when using the original features as inputs. However, the water/cement ratio is of great importance when predicting concrete compressive strength. To tackle this issue, other ML models are tested.

#### 3.2.2. Support Vector Regression (SVR)

Similar to the LR model, a linear SVR or kernelized SVR with polynomial kernel is not sufficient to make a better prediction. As mentioned in Section 2.2.2, Gaussian Radial basis function (RBF) kernel is able to map the original features into infinite dimensionality [46].

In this work, two hyperparameters, i.e., C and gamma (*γ*), are tuned. Among others, C is a hyperparameter controlling the balance between keeping the street as large as possible and limiting the margin violations: a smaller C leads to a wider street but more margin violation and vice versa. In other words, C acts as the regularization hyperparameter. Similarly, the kernel-related hyperparameter *γ* represents the effect of single sample on the hyperplane: The larger gamma is, the closer other examples must be to be affected and vice versa. Therefore, selecting proper value of C and *γ* is of great importance [47].

**Figure 4:**
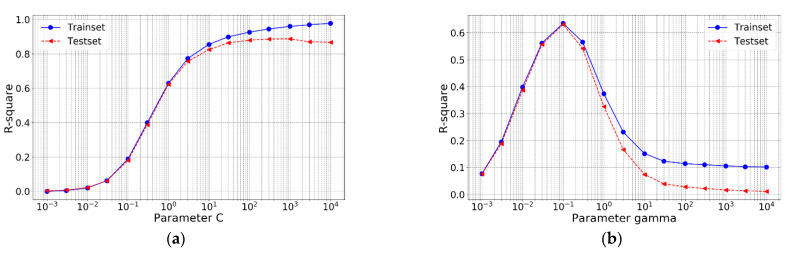
R-square of SVR under different hyperparameters: (**a**) different C and (**b**) different *γ*.

In order to roughly determine the search range, the possible optimal range of C and γ is first searched separately and the results are shown in Figure 4.

**Figure 5:**
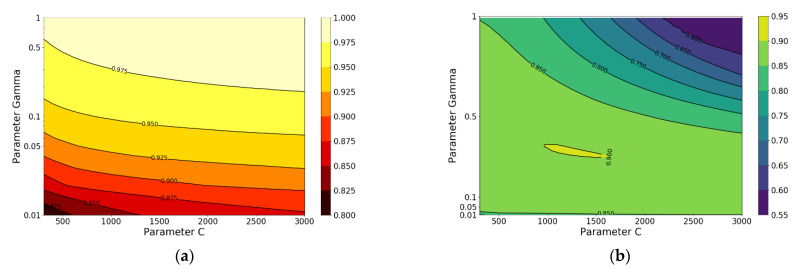
R-square of SVR under different hyperparameters C and *γ* on (**a**) training set and (**b**) test set.

From Figure 4, it is noted that when C equals to 1000 or γ equals to 0.1, the R-square of test set is greatest, respectively. Based on the obtained results, a grid search (C: change from 300 to 3000 with an increment of 100; *γ*: change from 0.01 to 1 with increment of 0.01) is carried out to look for the optimal combination of hyperparameters C and *γ* contributing to the relatively best performance of the model on test set. The R-squares of model in training set and test set under different hyperparameters C and *γ* are shown in Figure 5.

**Figure 6:**
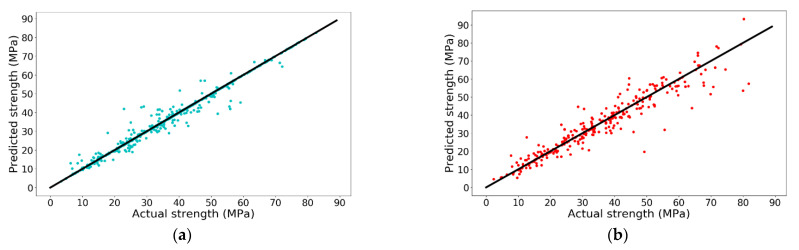
Prediction accuracy of SVR model on (**a**) training set and (**b**) test set.

From the obtained result, it can be concluded that when C and *γ* are 1300 and 0.32, respectively, the model has the best performance on the test set with an R-square value of 0.9006 and the accuracy is shown in Figure 6. It is obvious that the accuracy is higher than that of the best performing LR model (R-square = 0.8914).

#### 3.2.3. Extreme Gradient Boosting (XGBoost)

Although the performance of SVR model is better than the LR model, the prediction accuracy still has large space for improvement. Therefore, XGBoost, an ensemble method, is utilized to perform the regression. Before carrying out the prediction with XGBoost, we first import XGB model from the XGBoost package [48]. AS XGBoost is a scalable machine learning system for tree boosting, a lot of hyperparameters can be tuned. In this work, we only tune two hyperparameters: n_estimators and learning rate (shrinkage). The hyperparameter n_estimators represents the number of the base estimators and learning rate scales the contribution of each tree. The reason for choosing those two hyperparameters is that they have a relatively large influence on the state of the model compared to other hyperparameters.

**Figure 7:**
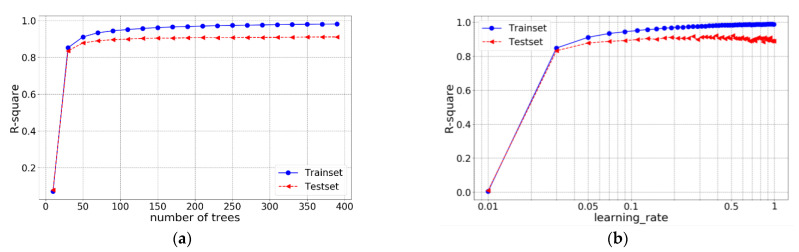
R-square of XGBoost under different hyperparameters: (**a**) different n_estimators; and (**b**) different learning rate.

Similar to the SVR model, grid search is employed to look for the optimal n_estimators and shrinkage. First, the possible optimal range of n_estimators and learning rate are identified separately and the results are shown in Figure 7.

**Figure 8:**
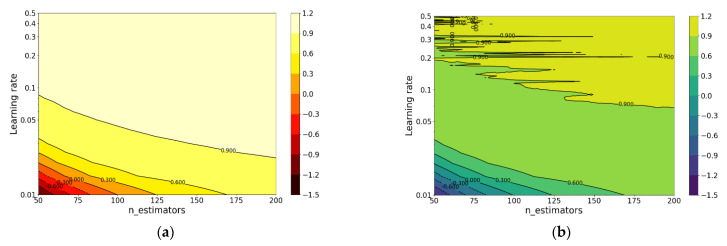
R-square of XGBoost under different n_estimators and learning_rate: (**a**) training set and (**b**) test set.

From the obtained result, when n_estimators equals 70 or learning rate equals 0.17, the R-square of the test set is greatest. Therefore, grid search (n_estimators: change from 50 to 200 with an increment of 1; learning rate: change from 0.01 to 0.5 with an increment of 0.005) is carried out to look for the optimal combination. The R-squares of XGBoost model in training set and test set under different hyperparameters are shown in Figure 8.

**Figure 9:**
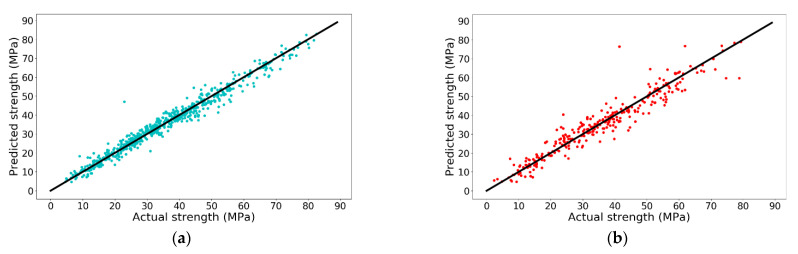
Prediction accuracy of XGBoost: (**a**) training set and (**b**) test set.

From the obtained result, when n_estimators and learning_rate are 64 and 0.365, respectively, the model has the best performance on the test set with an R-square of 0.9309 and the accuracy is shown in Figure 9.

**Figure 10:**
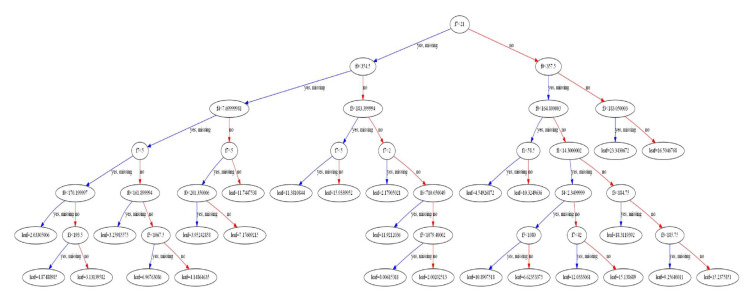
The 1st base estimator.

In addition, due to the white box property of XGBoost model, the base estimator could be drawn, which enables users to look into the regression process. For example, the first base estimator is shown in Figure 10.

**Figure 11:**
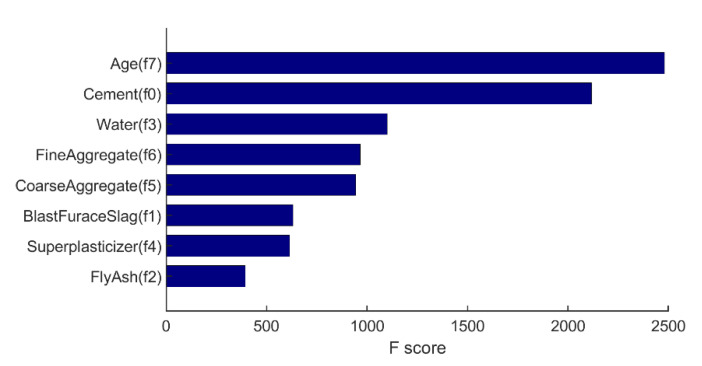
Feature importance of XGBoost model with original features.

Based on the results of all base estimators, the overall feature importance could also be obtained and the result is shown in Figure 11.

From Figure 11, it is noted that Age (f7) and cement content (f0) have the largest influence on the compressive strength, which is in accordance with the expectations. On the other hand, in this study, fly ash content (f2) has the least influence on the compressive strength compared to other features, which agrees with the result of Figure 10 where fly ash (f2) is not used for the regression. However, the result of XGBoost is influenced by many factors (random state, features, etc.), so the obtained feature importance only gives us an indication.

#### 3.2.4. Artificial Neural Network (ANN)

Last, ANN is adopted to predict the compressive strength. ANN is implemented using Keras [39] with Tensorflow backend. The batch size and the number of epochs are set to 100 and 1000, respectively. ReLu function is employed as the activation function; the Adam optimizer is used to improve the weights during the backpropagation process.

**Figure 12:**
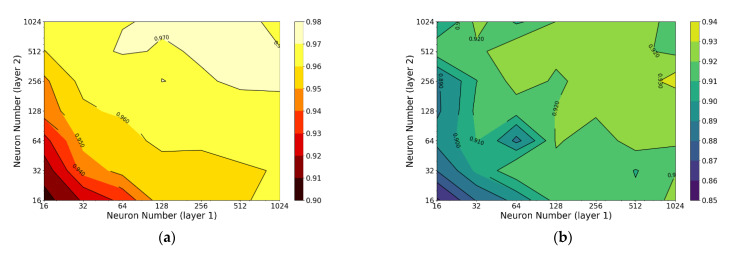
R-square of ANN with different number of neurons in each layer: (**a**) training set and (**b**) test set.

Similar to XGBoost, many hyperparameters could be tuned for ANN. In this work, hyperparameters related to neural network architecture are tuned first. Considering that the size of dataset is small, a 2-layer neural network is built and the numbers of neurons in each layer are tuned. The R-squares of model in training set and test set with different neuron numbers are shown in Figure 12.

**Figure 13:**
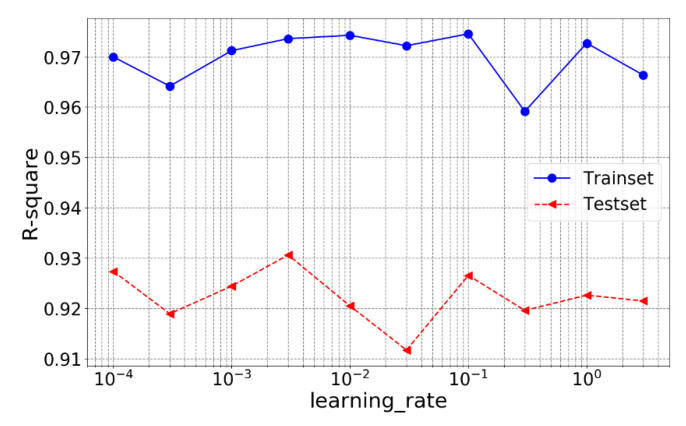
R-square of ANN with different learning rate.

From the obtained result, it can be concluded that when the neuron numbers of layer 1 and 2 are both 512, the model has the best performance on the test set with an R-square of 0.9324. After determining the number of the neurons in each layer, learning rate is also roughly tuned (selected values: 0.0001, 0.0003, 0.001, 0.003, 0.01, 0.03, 0.1, 0.3, 1, 3) and the result is shown in Figure 13.

**Figure 14:**
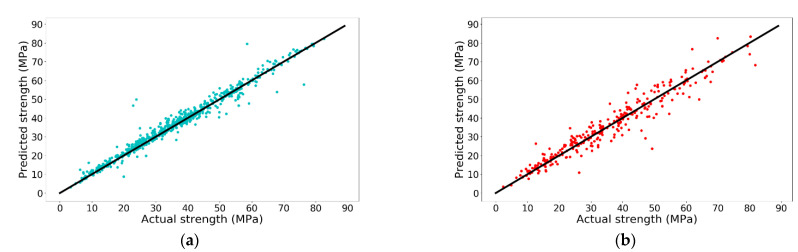
Prediction accuracy of ANN with different neurons in each layer: (**a**) training set and (**b**) test set.

From Figure 13, it is noted that when the learning rate is 0.003, the model has the best performance on the test set with an R-square of 0.9331, which is higher than the default learning rate. The accuracy of ANN with the tuned hyperparameters is shown in Figure 14.

### 3.3. Prediction Process with Manually Selected Features and PCA-Reduced Features

In Machine Learning, some problems may involve many features for each training instance. The large number of features not only makes training extremely slow, but it also makes harder to find a good solution. Dimensionality reduction is a good way to tackle this problem. In this paper, although the number of features is not large (i.e., 8), the performance of ML models with different number of features is investigated. On the one hand, the influence of reduced dimensionality on the accuracy could be compared with the model with the original features. On the other hand, the effectiveness of PCA in selecting the features and its influence on the four ML models are also studied.

Based on the result of PCA (see Section 3.1.1), when keeping the first six components of PCA-selected features, the cumulative explained variance is 0.9979, indicating that those features could well represent the original data without losing much information. Therefore, six PCA-selected features are used as the input to do the prediction. The relation between PCA-selected features and original features is obtained using SVD and shown in Equation (23).

(23) $${\left[\begin{array}{c} \mathrm{component}1\\ \mathrm{component}2\\ \mathrm{componnet}3\\ \mathrm{component}4\\ \mathrm{componnet}5\\ \mathrm{component}6 \end{array}\right]}^{T}\;=\;{\left[\begin{array}{c} \mathrm{cement}\\ \mathrm{BlastFurnaceSlag}\\ \mathrm{FlyAsh}\\ \mathrm{Water}\\ \mathrm{Superplasticizer}\\ \mathrm{CoaseAggregate}\\ \mathrm{FineAggregate}\\ \mathrm{Age} \end{array}\right]}^{T}\left[\begin{array}{cccccc} 0.906 & -0.033 & -0.155 & 0.008 & -0.151 & 0.307\\ -0.263 & -0.786 & -0.073 & 0.199 & -0.107 & 0.453\\ -0.239 & 0.303 & 0.052 & -0.687 & -0.178 & 0.512\\ 0.006 & -0.076 & 0.041 & -0.076 & 0.098 & -0.482\\ -0.001 & 0.005 & -0.024 & -0.021 & -0.023 & 0.104\\ -0.009 & 0.275 & 0.761 & 0.480 & -0.076 & 0.271\\ -0.210 & 0.451 & -0.611 & 0.485 & 0.133 & 0.257\\ -0.098 & -0.070 & 0.119 & -0.127 & 0.949 & 0.234 \end{array}\right]$$

In addition, six manually selected features are also used as input to compare with the other two scenarios. According to the previous studies [49], water to binder (W/B, Binder = cement+ fly ash+ blast furnace slag), fly ash to binder (FA/B), Blast Furnace slag to binder (BFS/B), superplasticizer to binder (SB), and sand ratio (SR) contribute to the compressive strength. Therefore, W/B, FA/B, BFS/B, SB, SR, and Age are chosen as the input to predict the compressive strength.

The prediction process with six PCA-selected features and six manually selected features is exactly the same as Section 3.2, so here only the results are shown. The results of 4 ML models with PCA-selected features and manually selected features are shown in Table 4 and Table 5, respectively.

## 4. Prediction Process with different ML Models

### 4.1. Performance on Training Set and Test Set

**Figure 15:**
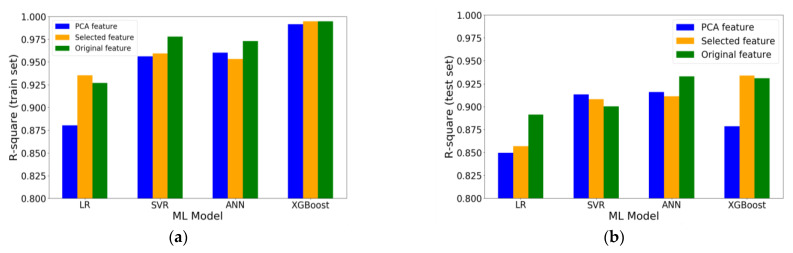
R-square of different ML models with different features: (**a**) training set and (**b**) test set.

**Figure 16:**
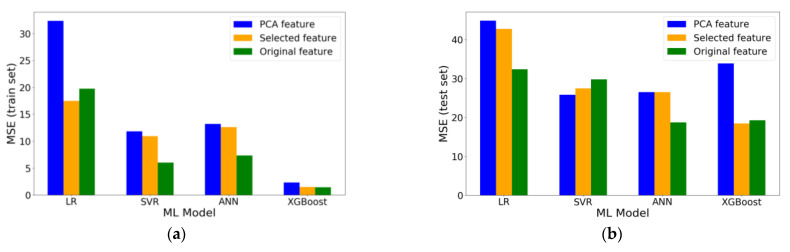
MSE of different ML models with different features: (**a**) training set and (**b**) test set.

After obtaining the prediction results from 4 ML models with three different features, a comprehensive comparison is made. First, the R-square and MSE of the training set and test set are investigated. The results are shown in Figure 15 and Figure 16.

From Figure 15, it is noted that the R-square of LR model on test set in all scenarios is less than 0.9, which is poorer than the other three ML models. For training set, except for the LR model, the R-squares with original features are the highest for each ML model. The reason why R-square of LR model with selected features is higher is that the polynomial degree of this model is 4, which is larger than the model with original features (i.e., 3). Among the ML models, XGBoost has the most admirable performance with R-square of over 0.99. The results are in good agreement with the trend of MSE in Figure 16a since a higher R-square corresponds to a smaller MSE.

As for test set, when using different features as inputs, the best performance occurs in different models (Figure 15b). Particularly, for original features, the model with highest R-square is ANN model (R-square = 0.9331); for PCA-selected features, the model with highest R-square is ANN model (R-square = 0.9160); for manually selected features, the model with highest R-square is XGBoost (R-square = 0.9339) and it is the best performance of all models. For the SVR model, the prediction accuracy of the model with manually selected features (R-square = 0.9080) or PCA-selected features (R-square = 0.9134) is better than the model with original features (R-square = 0.9003). In other words, dimensionality reduction has a positive influence on the prediction accuracy of SVR model.

Besides, it is worth mentioning that the R-square of XGBoost model with PCA-selected features in test set is only 0.8787, which is significantly lower than the other two ML models (0.9085 for ANN; 0.9134 for SVR), while still being higher than the LR model (R-square = 0.8496). A possible reason for this is that XGBoost model is a decision tree-based ML model. The prediction process of XGBoost is a white box and the features have a great influence on the overall performance of the model. From Equation (23), it is noted that the PCA-selected features do not address the influence of water to compressive strength as the weights for water are very low. Particularly, the weights of water from component 1–6 are 0.006, −0.076, 0.041, −0.076, 0.098, and −0.482, respectively. Clearly, this does not make sense because water is known to have great effect on compressive strength. Therefore, the prediction accuracy of XGBoost model with PCA-selected features is not admirable. In addition, according to feature analysis from Figure 11, the influence of fly ash is the least important, while in PCA-selected features, the weights of fly ash are relatively high (the weights of fly ash from component 1–6 are −0.239, 0.303, 0.052, −0.687, −0.178, and 0.512, respectively). In other words, the “important” features selected by PCA are not so important for the XGBoost Model.

**Figure 17:**
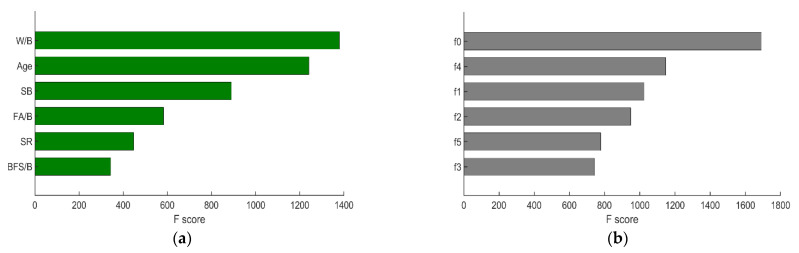
Feature importance of XGBoost model with 6 features: (**a**) manually selected features and (**b**) PCA-selected features.

On the other hand, it is inferred that distinguishable features are more likely to contribute to a better regression results. In order to verify the above inference, the feature importance of XGBoost model with PCA-selected features and manually selected features is drawn and shown in Figure 17.

From Figure 17, it is obvious that the PCA-selected features are not as distinguishable as the manually selected ones because the F scores of PCA-selected features are relatively concentrated. Therefore, it is inferred that, although the six PCA-selected features retain much of the original information, those features seem not to be as representative as the six manually selected ones. As a consequence, the performance of XGBoost with PCA-selected features is not as good as the one with manually-selected features. However, the prediction accuracy may be further improved by tuning other hyperparameters for XGBoost, which should be studied in the future.

### 4.2. Training Speed

**Figure 18:**
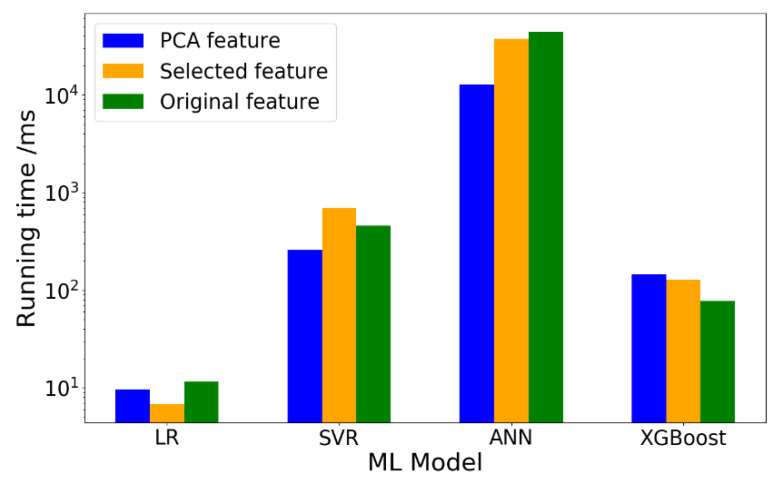
Running time of trainset under different ML model.

Except for the performance of ML models in terms of prediction accuracy, another important issue is the training speed. In practice, if the computing speed of a ML model is fast, more time can be devoted to hyperparameter tuning to improve the performance of the ML model. Generally, the computing speed is related to the algorithm itself and the number of features. Considering that the running time of the same ML model may largely vary in different time, the average running time, which is calculated as the average running time of the ML model, ran 10 times. The running time of four ML models with different features is shown in Figure 18.

Based on the results from Figure 18, it is noted that the running time of ANN model is the longest while that of LR model is the shortest. However, the running time of ANN model with PCA-selected features (t = 12.78s) is much shorter than the model with original features (t = 44.28s). Considering that the prediction accuracy gap between ANN models with original features and PCA-selected features is not too large (0.017), it can be concluded that dimensionality reduction has an obviously positive influence on running time without losing much prediction accuracy for ANN model.

In addition, it is noted that the running time of XGBoost model with PCA-selected features is even longer than XGBoost model with original model or manually selected features. Therefore, in this study, dimensionality reduction by PCA has an adverse effect both on the performance and the running time of XGBoost model.

## 5. Conclusions

In this work, the prediction of concrete compressive strength based on given features is carried out with four representative ML models. In addition, the performances of those ML models with different features are compared. Meanwhile, the training speed of the four ML models is also investigated. Based on the presented results, the following conclusions can be drawn:

1. Among the four ML models, linear regression has the poorest performance with an R-square of less than 0.90, while the other 3 ML models have an R-square of over 0.90. Therefore, it is possible to make an accurate prediction of the compressive strength using some ML models such as Support Vector Regression (SVR), Extreme Gradient Boosting (XGBoost), and Artificial Neural Network (ANN).
2. The highest R-square of test set of ML model with original features, PCA-selected features and manually selected features are 0.9331 (ANN), 0.9160 (ANN), and 0.9339 (XGBoost) respectively. Therefore, the performance of XGBoost model with manually selected features is the model with the best performance of all models.
3. The prediction accuracy of SVR model with manually selected features (R-square = 0.9080) or PCA-selected features (R-square = 0.9134) is better than the model with original features (R-square = 0.9003) without dramatic running time change, indicating that dimensionality reduction has an admirable influence on SVR model.
4. Compared with the XGBoost model with original features and manually selected features, the model with PCA-selected features has a relatively poorer performance (R-square = 0.8787). The possible reason for this is inferred that the PCA-selected features are not as distinguishable as the manually selected features in this study.
5. The running time of XGBoost model with PCA-selected features or manually selected features is longer than XGBoost model with original features. In this work, dimensionality reduction by PCA seems to have an adverse effect both on the performance and the running time for XGBoost model.
6. Although the running time of ANN model is much longer than the other three models (less than 1s) in 3 scenarios, dimensionality reduction has an obviously positive influence on running time without losing much prediction accuracy for ANN model.

## Figures and Tables

**Table 1:** Statistical properties of Yeh’s dataset [28].

	Cement (kg/m^3^)	Blast Furnace Slag (kg/m^3^)	Fly Ash (kg/m^3^)	Water (kg/m^3^)	Superplasticizer (kg/m^3^)	Coarse Aggregate (kg/m^3^)	Fine Aggregate (kg/m^3^)	Age (day)	Compressive Strength (MPa)
Mean	281.168	73.896	54.188	181.567	6.205	972.919	773.580	45.662	35.818
Std	104.456	86.237	63.966	21.344	5.971	77.716	80.137	63.139	16.698
Min	102	0	0	121.8	0	801	594	1	2.33
Max	540	359.4	200.1	247	32.2	1145	992.6	365	82.6
Range	358	359.4	200.1	125.2	32.2	344	398.6	364	80.26

**Table 2:** Result of Principal Component Analysis (PCA).

Component	Component 1	Component 2	Component 3	Component 4	Component 5	Component 6	Component 7	Component 8
Explained variance ratio	3.2577 × 10^−1^	2.4887 × 10^−1^	1.8480 × 10^−1^	1.0766 × 10^−1^	1.0095 × 10^−1^	2.9845 × 10^−2^	1.8180 × 10^−3^	2.8770 × 10^−4^
Cumulative explained variance	0.32577	0.57464	0.75944	0.86710	0.96805	0.997895	0.999713	1.00000

**Table 3:** Result of LR model with different polynomial features.

Degree	Dimensionality of New Features	R-Square	
		Training Set	Test Set
1	8	0.6133	0.6162
2	44	0.8114	0.7916
3	164	0.9269	0.8914
4	494	0.9834	−596.344

**Table 4:** Prediction results with 6 PCA-selected features.

ML Model	R-Square		MSE		Hyperparameters
	Trainset	Testset	Trainset	Testset	
LR	0.8803	0.8496	32.3600	44.9011	Polynomial Degree = 3
SVR	0.9563	0.9134	11.8244	25.8550	C = 550
					γ = 0.23
XGBoost	0.9916	0.8787	2.3397	33.8782	n_estimators = 190
					learning_rate = 0.07
ANN	0.9602	0.9160	13.2096	26.4843	#neuron = 128 (layer1)
					#neuron = 128 (layer2) learning_rate = 0.0003

**Table 5:** Prediction results with 6 manually selected features.

ML Model	R-Square		MSE		Hyperparameters
	Trainset	Testset	Trainset	Testset	
LR	0.8803	0.8496	32.3600	44.9011	Polynomial Degree = 4
SVR	0.9563	0.9134	11.8244	25.8550	C = 2000
					γ = 0.27
XGBoost	0.9916	0.8787	2.3397	33.8782	n_estimators = 140
					learning_rate = 0.23
ANN	0.9602	0.9160	13.2096	26.4843	#neuron = 512 (layer1)
					#neuron = 512 (layer2) learning_rate = 0.1

## Data Availability

Data sharing is not applicable to this article.
